# Genetically predicted levels of folate, vitamin B_12_, and risk of autoimmune diseases: A Mendelian randomization study

**DOI:** 10.3389/fimmu.2023.1139799

**Published:** 2023-03-10

**Authors:** Hong Yang, Jie Song, Aole Li, Linshuoshuo Lv, Xiaohui Sun, Yingying Mao, Ding Ye

**Affiliations:** ^1^ Department of Epidemiology, School of Public Health, Zhejiang Chinese Medical University, Hangzhou, China; ^2^ The Fourth College of Clinical Medicine, Zhejiang Chinese Medical University, Hangzhou, China

**Keywords:** folate, vitamin B_12_, autoimmune diseases, Mendelian randomization, single nucleotide polymorphism

## Abstract

**Background:**

Evidence from observational studies on the association of folate and vitamin B_12_ with autoimmune diseases are conflicting.

**Objective:**

We aimed to investigate the relationship of folate and vitamin B_12_ with autoimmune diseases using Mendelian randomization (MR).

**Materials and methods:**

We selected single-nucleotide polymorphisms associated with folate and vitamin B_12_ at the genome-wide significance level. Summary-level data for four common autoimmune diseases (vitiligo, inflammatory bowel disease, rheumatoid arthritis, and systemic lupus erythematosus) were obtained from large-scale genome-wide association studies, with a sample size of 44,266, 86,640, 58,284, and 23,210, respectively. MR analyses were conducted using the inverse variance weighted (IVW) approach, and sensitivity analyses were further performed to test the robustness.

**Results:**

We found that a higher genetically determined serum folate level per one standard deviation (SD) was associated with a decreased risk of vitiligo by the IVW method [odds ratios (OR) = 0.47; 95% confidence interval (CI): 0.32–0.69; *P* = 1.33 × 10^-4^]. Sensitivity analyses using alternative methods showed similar associations, and no evidence of pleiotropy was detected by MR-Egger regression (*P* = 0.919). In addition, we observed that vitamin B_12_ per one SD was positively associated with IBD (IVW: OR = 1.14, 95% CI: 1.03–1.26, *P* = 0.010; maximum likelihood: OR = 1.14, 95% CI: 1.01–1.29, *P* = 0.035; MR-PRESSO: OR = 1.14, 95% CI:1.01–1.28, *P* =0.037), while the association was not significant after Bonferroni correction.

**Conclusion:**

The study provides convincing evidence for an inverse association between serum folate level and risk of vitiligo. Further studies are warranted to elucidate the possible association between vitamin B_12_ and risk of IBD.

## Introduction

Autoimmune diseases represent a family of illnesses that share a common pathogenesis of an immune-mediated attack on the body’s own organs (1). Accumulating epidemiological studies have shown that autoimmune diseases have caused a serious disease burden on the world—for example, the global prevalence of vitiligo is 0.06%–8.80% according to research published between 1964 and 2017 (2). The Global Burden of Disease study reported that (3) there were 4.90 million prevalent cases of inflammatory bowel disease (IBD) in 2019, resulting in 41,000 deaths and 1.62 million disability adjusted life years (DALYs) in global. Moreover, autoimmune diseases may have serious tissue damage before clinical diagnosis and due to lack of effective treatment (1)—for instance, the hazard of systemic lupus erythematosus (SLE) may involve multiple organs, including the kidneys, skin, joints, and nervous system (4). Rheumatoid arthritis (RA) can cause cartilage and bone damage as well as disability (5). IBD is a chronic inflammatory disorder primarily of, but not restricted to, the gut because extraintestinal manifestations are frequently observed in joints, eyes, hepatobiliary tract, and skin (6).

Folate and vitamin B_12_ are essential water-soluble vitamins that play a crucial role in the maintenance of one-carbon metabolism. B vitamins control DNA synthesis and methyl donor availability, which are important for the normal function of cells in the immune system (7). Notwithstanding, the association of folate and vitamin B_12_ with autoimmune diseases is not fully understood. Recently, a meta-analysis involving a total of 804 patients with vitiligo and 518 controls indicated that vitiligo patients had lower vitamin B_12_ levels [standardized mean difference (SMD) -0.430; 95% CI, -0.738 to -0.121] than controls, while there was no significant difference in the serum folate level between 672 patients and 378 matched controls (8). Similarly, Tsung-Yu Tsai et al. conducted a meta-analysis of eight studies involving 415 patients with SLE and 391 controls which showed that SLE patients had significantly lower vitamin B_12_ levels than the controls (SMD, -0.359; 95% CI, -0.638 to -0.080), but the serum folate level did not differ markedly between 532 SLE patients and 634 controls (9). For IBD, a meta-analysis including 1,086 IBD patients and 1,484 controls showed that the serum folate concentration in IBD patients was significantly lower than that in control patients (SMD, -0.46; 95% CI, -0.64 to -0.27), whereas the mean serum vitamin B_12_ concentration did not differ between cases and controls (10). Therefore, the association of folate and vitamin B_12_ with autoimmune diseases was still debated. Moreover, these associations derived from traditional observational epidemiological studies are susceptible to confounders and reverse causation bias.

Mendelian randomization (MR) is a genetic epidemiological method which uses genetic variants, especially single-nucleotide polymorphisms (SNPs), as instrumental variables (IVs) to infer the potential causality of exposure and outcome (11). The MR approach can overcome confounders and reverse causation bias as the genotype of an individual is determined at conception and cannot be changed. To the best of our knowledge, there are no MR studies inferring the potential causal relationship of folate and B_12_ with these four autoimmune diseases (vitiligo, IBD, RA, and SLE) to date. Therefore, we applied the two-sample MR method to examine whether the genetically predicted folate and B_12_ levels were associated with the risk of autoimmune diseases.

## Methods

**Figure 1:**
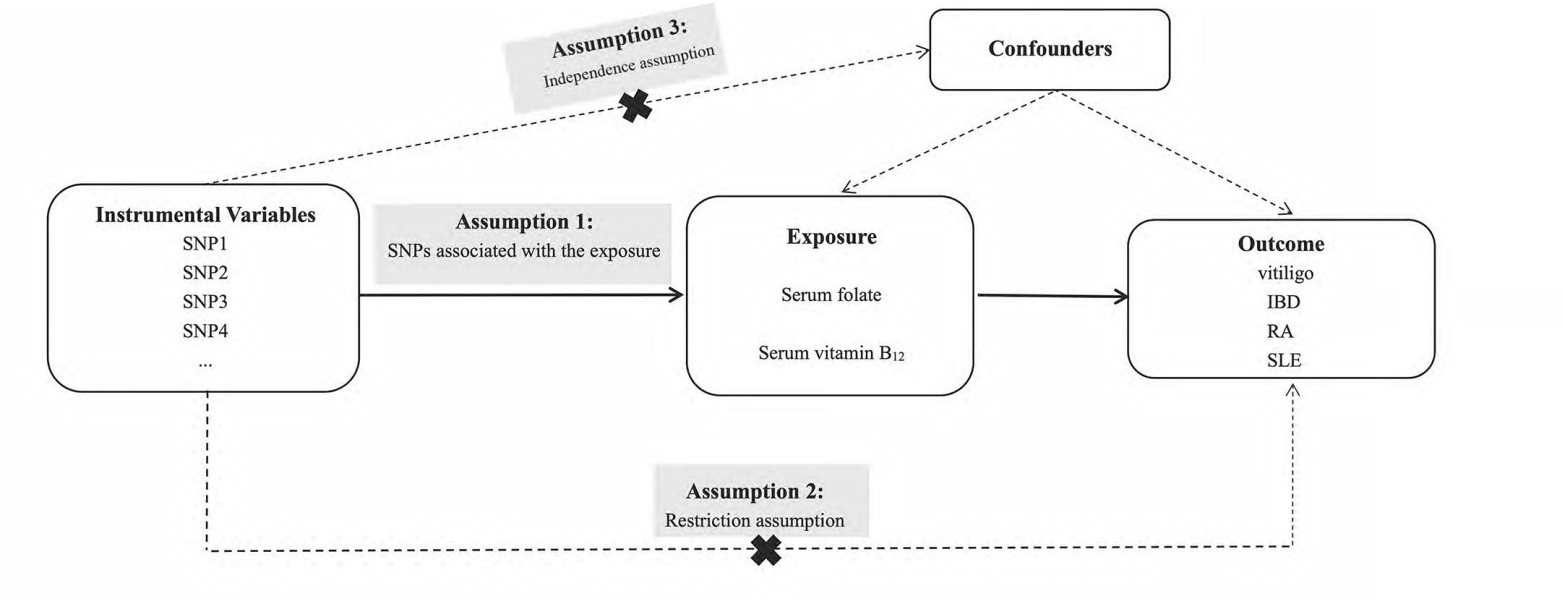
Overall design of Mendelian randomization analyses in the present study.

The overview of our study design is shown in **Figure 1**. Since the data that we used were based on published studies and public databases, no additional ethical approval from an institutional review board was required.

### Outcome source

We gathered the summary statistics of autoimmune diseases from published genome-wide association studies (GWASs). To be more specific, the sample sizes of datasets for vitiligo (12), IBD (13), RA (14), and SLE (15) are 44,266 (4,680 cases), 86,640 (38,155 cases), 58,284 (14,361 cases), and 23,210 (7,219 cases), respectively. All the participants were of European ancestry. The detailed information of the outcome GWASs is listed in **Supplementary Table S1**.

### Selection of instrumental variable

Genetic variants for folate and vitamin B_12_ levels were derived from a GWAS including 37,341 and 45,576 European individuals, respectively (16). The population involved in the GWAS were from Iceland and Denmark. Specifically, the sample size of GWASs for folate and B_12_ were 28,913 and 37,283 from Iceland and 8,428 and 8,293 from Denmark, respectively. A total of four and 15 SNPs at the genome-wide significance level (*P* < 5 × 10^-8^) were selected as IVs for folate and vitamin B_12_ in primary analysis (**Supplementary Table S2**). Furthermore, we excluded two SNPs for folate with linkage disequilibrium (LD) (*r* ^2^ threshold < 0.1) through the Web source (https://snipa.helmholtzmuenchen.de/snipa3/index.php?task=pairwise_ld) in the sensitivity analysis, and no LD was found among the 15 instrumental SNPs for vitamin B_12_.

Then, we calculated the *F*-statistics; to quantify the strength of the IVs that we used to assess weak instrumental variable bias, the *F*-statistic for folate and vitamin B_12_ ranged from 22.05 to 568.86 (**Supplementary Table S3**), satisfying the assumption of *F >*10 for MR analyses (17). The variance explained by the IVs for folate and vitamin B_12_ with different outcomes was approximately 1.1% and at least 2.7% (**Supplementary Table S3**). Furthermore, we detected the minimum effect size based on 80% statistical power at an *α* level of 5% using an online tool (https://shiny.cnsgenomics.com/mRnd/). Based on the sample size of outcome datasets, there was sufficient power to detect significant associations with any autoimmune diseases for folate and vitamin B_12_ at an effect size (odds ratio, OR) of 1.64 and 1.38 (**Supplementary Table S3**). Then, we scanned other relevant phenotypes of the selected IVs through the GWAS catalog (https://www.ebi.ac.uk/gwas/, accessed on July 1, 2022) to further certify the impact of horizontal pleiotropy on causality effect (**Supplementary Table S4**).

### Statistical analysis

We conducted a two-sample Mendelian randomization analysis to estimate the potential causal relationship of folate and vitamin B_12_ with different autoimmune diseases using the “Mendelian randomization” and “MRPRESSO” packages in R software version 3.6.3. The MR study was based on three core assumptions: (1) there is a robust and strong association between IVs and exposure; (2) the IVs are independent of confounders; and (3) the IVs can only affect the outcome through exposure without other pathways. We mainly used inverse variance weighted (IVW) method for MR analysis, which provided a consistent estimate of the association between exposure and the risk of outcomes when IVs were not pleiotropic (18). Cochran’s Q statistics was performed to assess the heterogeneity across individual SNPs. The fixed-effects model was employed if no substantial heterogeneity (*P* < 0.05) was observed; otherwise, the random-effects model was utilized (19). We also performed sensitivity analyses to verify the robustness of our results. The weighted median method can obtain a consistent estimate of the overall effect when more than 50% of IVs are valid, which can reduce the bias of the causal effect estimate compared with the IVW method (20). The maximum-likelihood method may provide more reliable estimates when measurement error occurs in the SNP-exposure effects (21). Moreover, the MR-Egger method was used to determine whether the instrumental SNPs are multi-effect. The intercept obtained from the MR-Egger regression was used to measure the directional pleiotropy (22). In addition, we used MR pleiotropy residual sum and outlier test (MR-PRESSO) in order to detect and correct for multi-effects by removing possible outliers (18).

Statistical significance was set at a *P*-value <6.25 × 10^-3^ (*P* = 0.05/(two exposures × four outcomes) corrected for two exposures and four outcomes using the Bonferroni method. A *P*-value above the Bonferroni-corrected threshold, but lower than 0.05, was considered suggestive evidence for a potential causal association. All statistical analyses were two-sided.

## Results

**Figure 2:**
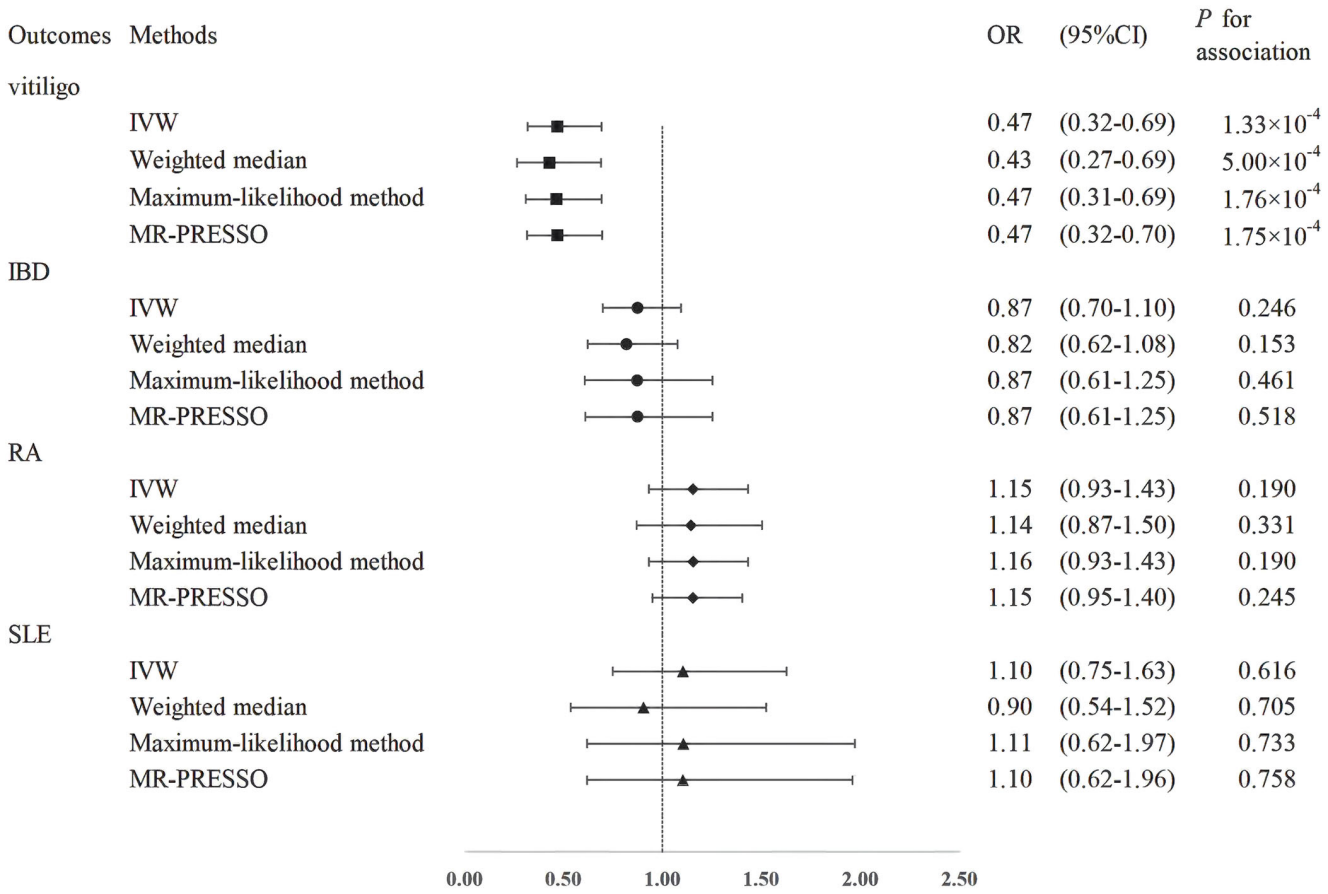
Mendelian randomization estimates from instrument variants for folate on risk of autoimmune diseases.

**Figure 3:**
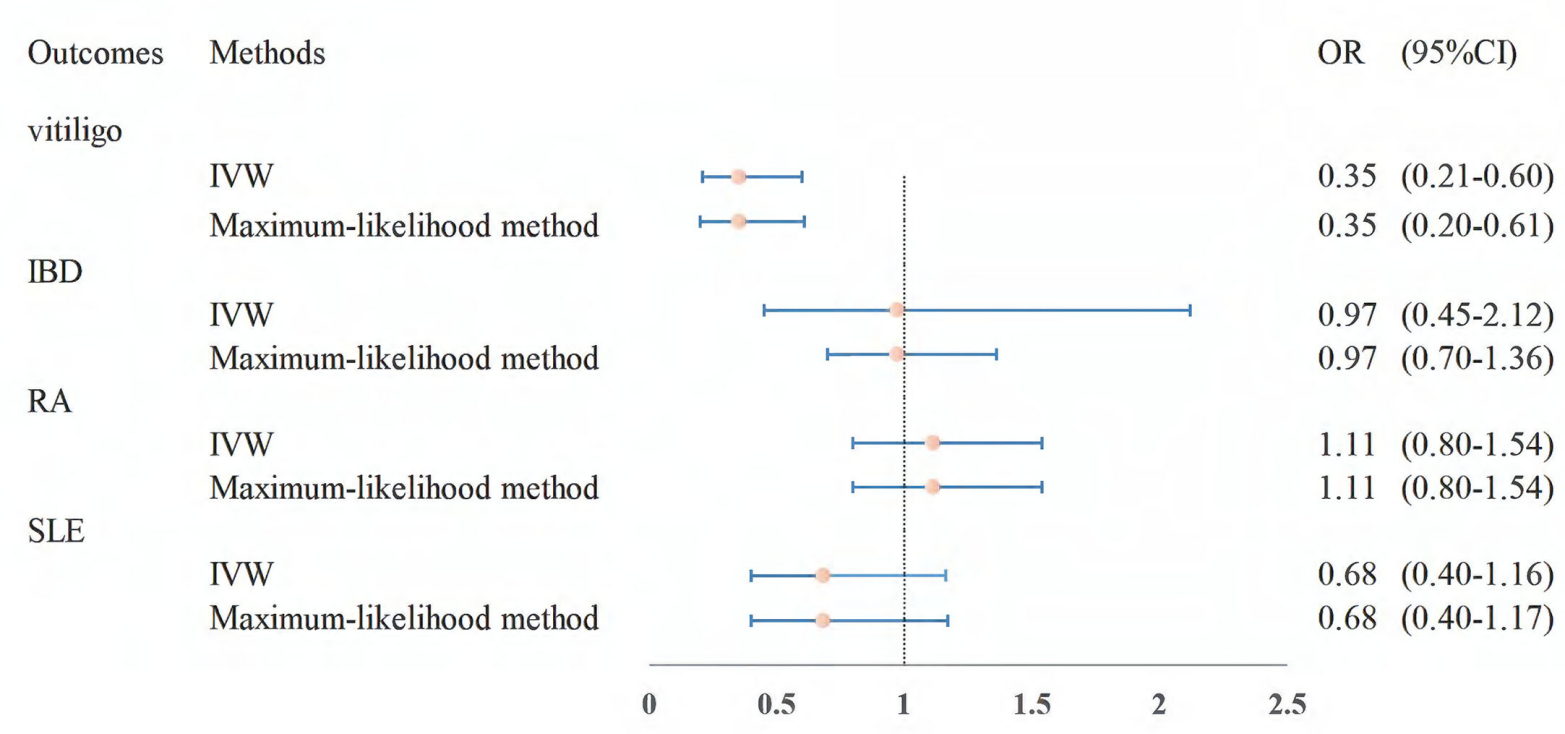
MR estimates from instrument variants for folate on risk of autoimmune diseases after removing SNPs with LD. MR, Mendelian randomization; SNP, single-nucleotide polymorphism; LD, linkage disequilibrium.

The effect estimates of the MR analyses for the association of folate with four autoimmune diseases are depicted in **Figure 2**. There was no evidence for heterogeneity (vitiligo: *P* = 0.382, IBD: *P* = 0.056; RA: *P* = 0.480; SLE: *P* = 0.085) by using Cochran’s Q test, so we chose the fixed-effect IVW model for MR analysis. We observed that a higher genetically predicted serum folate level per one standard deviation (SD) was significantly associated with a decreased risk of vitiligo by the IVW method (OR = 0.47; 95% CI: 0.32–0.69; *P* =1.33 × 10^-4^). The sensitivity analysis showed similar results (OR: 0.43, 95% CI: 0.27–0.69, *P* = 5.00 × 10^-4^ by weighted median; OR: 0.47, 95% CI: 0.31–0.69, *P* = 1.76 × 10^-4^ by maximum-likelihood method; OR: 0.47, 95% CI: 0.32–0.70, *P* = 1.75 × 10^-4^ by MR-PRESSO). Moreover, MR-Egger regression did not suggest evidence of potential directional pleiotropy (*P* for intercept = 0.919). In addition, we found that the direction and effect of the association between folate and vitiligo remained consistent after excluding the two SNPs with LD (**Figure 3**). The IVW method showed that the genetically predicted serum folate level per one SD increment was associated with a decreased risk of vitiligo (OR = 0.35, 95% CI: 0.21–0.60, *P* = 1.47×10^-4^). Moreover, the results were stable by maximum-likelihood method (OR = 0.35, 95% CI: 0.20–0.61, *P* = 2.12 × 10^-4^). Here we could not use the MR-Egger method to determine whether the SNPs are horizontally pleiotropic due to the fact that the MR-Egger method required at least three SNPs. Similarly, we cannot detect outlier SNPs by the MR-PRESSO method due to lack of enough SNPs. We further performed a leave-one-out analysis to detect whether the causal estimate was driven by any single SNP, which revealed a consistent inverse association between genetically predicted folate and risk of vitiligo (**Supplementary Figure S1**).

However, the results showed no statistically significant associations of folate level with other autoimmune diseases (IBD: OR = 0.87, 95% CI: 0.70–1.10, *P* = 0.246; RA: OR = 1.16, 95% CI: 0.93–1.43, *P* = 0.190; SLE: OR = 1.10, 95% CI: 0.75–1.63, *P* = 0.616; **Figure 2**). The null findings were supported by the weighted median method, the maximum-likelihood method, and MR-PRESSO. There was also no apparent sign of horizontal pleiotropy as assessed by MR-Egger regression (*P*-values of the intercept for IBD: *P* = 0.670; RA: *P* = 0.469; SLE: *P* = 0.891). Furthermore, we observed that the results were stable in the sensitivity analysis after excluding the two SNPs with LD (**Figure 3**).

For vitamin B_12_, the IVW method showed that the genetically predicted vitamin B_12_ level per one SD increment may be associated with a 14% increased risk of IBD (95% CI: 3%–26%, *P* = 0.010), but alternative MR methods showed wider CIs (**Table 1**). However, all these associations did not meet the statistical test following multiple testing correction (*P*=6.25×10^-3^). In the meanwhile, we did not detect any associations of vitamin B_12_ with other autoimmune diseases (**Table 1**). Similarly, there was no apparent sign of horizontal pleiotropy as assessed by MR-Egger regression (vitiligo: *P* = 0.819; IBD: *P* = 0.196; RA: *P* = 0.615; SLE: *P* = 0.914).

**Table 1:** Mendelian randomization (MR) analysis of vitamin B_12_ with autoimmune disease risk.

Risk factors	Number of single-nucleotide polymorphism s	OR	95% CI		P for association	*P* for MR-Egger intercept	*P* for heterogeneity test
B_12_ and vitiligo							
Inverse-variance weighted (fixed)	12	1.01	0.83	1.22	0.934		0.867
MR-Egger	12	1.05	0.70	1.58	0.809	0.819	
Weighted median	12	1.02	0.80	1.32	0.857		
Maximum-likelihood method	12	1.01	0.83	1.22	0.934		
MR-PRESSO	12	1.01	0.88	1.16	0.913		
B_12_ and inflammatory bowel disease							
Inverse-variance weighted (fixed)	14	1.14	1.03	1.25	0.010		0.101
MR-Egger	14	1.28	1.03	1.57	0.023	0.196	
Weighted median	14	1.14	0.98	1.32	0.087		
Maximum likelihood	14	1.14	1.01	1.29	0.035		
MR-PRESSO	14	1.14	1.01	1.28	0.037		
B_12_ and rheumatoid arthritis							
Inverse-variance weighted (fixed)	11	1.01	0.89	1.15	0.851		0.299
MR-Egger	11	1.08	0.80	1.47	0.601	0.615	
Weighted median	11	1.02	0.87	1.20	0.811		
Maximum-likelihood method	11	1.01	0.88	1.16	0.862		
MR-PRESSO	11	1.01	0.88	1.16	0.866		
B_12_ and systemic lupus erythematosus							
Inverse-variance weighted (random)	12	0.99	0.76	1.27	0.908		0.013
MR-Egger	12	0.96	0.56	1.65	0.882	0.914	
Weighted median	12	1.13	0.88	1.47	0.337		
Maximum-likelihood method	12	0.99	0.76	1.27	0.911		
MR-PRESSO (outlier corrected)	11	1.19	1.01	1.41	0.069		

## Discussion

Research on preventing autoimmune diseases through a nutritious diet has gradually become attractive because diet is easier to correct than the other modifiable unhealthy lifestyles. However, to the best of our knowledge, randomized controlled trial (RCT) studies on folate and vitamin B_12_ with autoimmune diseases are rare to date. Our MR study found a significant association of one SD higher genetically predicted folate level with a decreased risk of vitiligo, providing suggestive evidence as well of the potential causal relationship between vitamin B_12_ and risk of IBD.

Vitiligo is an autoimmune disease which destroys skin melanocytes and gives the appearance of patchy depigmentation (23). The aforementioned meta-analysis (8) including nine previous observational studies showed that folate may be not associated with a decreased risk of vitiligo. However, the studies were heterogeneous due to sample size and ethnic differences among the vitiligo patients. Moreover, the included observational studies were eight case–control studies and one cross-sectional study, which is insufficient to prove the causal association (8). The directionality of the MR association between genetically determined levels of folate and risk of vitiligo is consistent with experimental data showing a preventive role for folate in vitiligo. It is well known that folate plays a role in the transformation of homocysteine (Hcy), which could lower the intracellular Hcy level (24). A recent study published by Chen et al. reported that Hcy could induce melanocyte apoptosis *via* the PERK-eIF2α-CHOP pathway in vitiligo patients and provide evidence that folate supplementation can reverse the melanin production defects in melanocytes induced by Hcy. Folate can protect melanocytes from Hcy-induced apoptosis and inhibition of melanin synthesis (24). In addition, folate is implicated in the biosynthesis of melanin in human epidermis because it is necessary for the production of tetrahydrobiopterin in melanocytes and keratinocytes (25–27). Tetrahydrobiopterin regulates the tyrosinase activity in melanosome, and the synthesized tetrahydrobiopterin is highly activated in vitiligo (28, 29). Although the explanation of the association between folate and the risk of vitiligo was biologically reasonable, more macro and micro evidence such as related preventive RCTs and animal experimental studies is warranted to elucidate the mechanism.

In a previous MR study on the environmental risk factors for IBD with the outcome dataset including 25,042 IBD cases and 34,915 controls, one SD increase in circulating vitamin B_12_ was not significantly associated with IBD (OR = 1.07, 95% CI: 1.00–1.15, *P* = 0.06) (30). Our well-powered MR study with a larger sample size found that there was suggestive evidence of a potential hazard effect of vitamin B_12_ on the risk of IBD, although the association did not reach statistical significance after correction for multiple comparisons. Vitamin B_12_ is bound to dietary protein and absorbed after the hydrochloric acid produced in the gastric mucosa separates it from the protein. The released cobalamin then attaches to the R protein and enters into the duodenum, where the R protein is removed and the free cobalamin binds to the intrinsic factor (IF). The IF–cobalamin complex is absorbed by the distal ileum and requires calcium (31). The former studies suggested that the absorption mechanisms of vitamin B_12_ could reach saturation (32, 33); thus, excessive B_12_ levels may burden the gut absorption function. A randomized double-blind placebo-controlled trial concluded that surplus treatment with vitamin B_12_ in IBD patients has no beneficial clinical effect (34). In addition, an animal study reported that vitamin B_12_ deficiency protects against DSS-induced colitis in mice (35), which confirms that higher B_12_ levels may increase the risk of IBD.

For RA, there were limited observational studies on the association of folate and vitamin B_12_ with risk of RA to date. Our study found null associations of folate and vitamin B_12_ concentrations with RA risk, which suggested that folate and vitamin B_12_ supplement may not be beneficial for preventing from RA, thus clinicians should use nutritional supplements with caution in practice. Although there was a meta-analysis showing a protective effect of folate on SLE and no marked difference in the folate level of patients with SLE and controls, the including studies were all of case-control studies (9), which are insufficient to prove the causal association due to flaws such as confounders and reverse causation bias in case-control studies. As compared with previous findings, our MR results are more convincing since it avoids confounders and reverse causal association bias to some extent. Consequently, we found that folate and B_12_ were not significantly associated with SLE.

However, the present study has some limitations. First, the validity of the MR study may be reduced due to the weak IVs. To remedy this problem, we used independent SNPs reaching genome-wide significance level (*P* < 5 × 10^-8^), and the *F*-statistic satisfied the threshold of >10. However, a larger sample size of GWAS is warranted to find more folate-related SNPs in the future. Second, pleiotropy may occur when some instrumental SNPs are associated with other multiple factors. In the present study, we did not observe evidence of pleiotropy for the causal association between folate and risk of vitiligo in any of the above-mentioned MR approaches. Third, since the subjects are limited to Europeans, the results may not be applicable to the entire population, and the research conclusions should be extrapolated cautiously. Fourth, we could not investigate the nonlinear effects of folate and vitamin B_12_ on these autoimmune diseases due to the utilization of summary-data level in two-sample MR analysis. Nonetheless, our study offers new insights into the relationships between the folate level and vitiligo, providing a better understanding of its etiology.

## Conclusion

In summary, genetically determined higher circulating levels of folate were associated with a decreased risk of vitiligo using MR methods. Our research suggests that folate supplementation may be a potential preventive measure for vitiligo, which provides a rationale for using folate supplementation as a promising target for vitiligo prevention. Further studies are needed to determine how folate affects the occurrence and development of vitiligo and confirm the association in large-scale randomized controlled trials and scientific animal experiments.

## Data availability statement

Datasets used for the MR analyses are public and available under reasonable requests. The authors thank all investigators for sharing these data.

## Ethics statement

Since the data we used were based on published studies and public databases, no additional ethical approval from an institutional review board was required. The patients/participants provided their written informed consent to participate in this study.

## Author contributions

HY conducted the data analysis and drafted the manuscript. JS participated in mining the database and drafting the manuscript. AL and LL searched literatures and analyzed the data. XS took responsibility of the tables and figures of the results. DY and YM designed the study, revised the manuscript, and provided technical support. All authors contributed to the article and approved the submitted version.

## References

[1] Rose NR. Prediction and prevention of autoimmune disease in the 21st century: A review and preview. Am J Epidemiol (2016) 183(5):403–6. doi: 10.1093/aje/kwv292 26888748

[2] Said-Fernandez SL Sanchez-Domínguez CN Salinas-Santander MA Martinez-Rodriguez HG Kubelis-Lopez DE Zapata-Salazar NA. Novel immunological and genetic factors associated with vitiligo: A review. Exp Ther Med (2021) 21(4):312. doi: 10.3892/etm.2021.9743 33717255 PMC7885061

[3] Diseases GBD Injuries C. Global burden of 369 diseases and injuries in 204 countries and territories, 1990-2019: A systematic analysis for the global burden of disease study 2019. Lancet (2020) 396(10258):1204–22. doi: 10.1016/S0140-6736(20)30925-9 PMC7567026 33069326

[4] Durcan L O'Dwyer T Petri M. Management strategies and future directions for systemic lupus erythematosus in adults. Lancet (2019) 393(10188):2332–43. doi: 10.1016/S0140-6736(19)30237-5 31180030

[5] Smolen JS Aletaha D McInnes IB. Rheumatoid arthritis. Lancet (2016) 388(10055):2023–38. doi: 10.1016/s0140-6736(16)30173-8 27156434

[6] Greuter T Vavricka SR. Extraintestinal manifestations in inflammatory bowel disease - epidemiology, genetics, and pathogenesis. Expert Rev Gastroenterol Hepatol (2019) 13(4):307–17. doi: 10.1080/17474124.2019.1574569 30791773

[7] Lyon P Strippoli V Fang B Cimmino L. B vitamins and one-carbon metabolism: Implications in human health and disease. Nutrients (2020) 12(9):2867. doi: 10.3390/nu12092867 PMC7551072 32961717

[8] Tsai TY Kuo CY Huang YC. Serum homocysteine, folate, and vitamin B(12) levels in patients with vitiligo and their potential roles as disease activity biomarkers: A systematic review and meta-analysis. J Am Acad Dermatol (2019) 80(3):646–54.e5. doi: 10.1016/j.jaad.2018.08.029 30165163

[9] Tsai TY Lee TH Wang HH Yang TH Chang IJ Huang YC. Serum homocysteine, folate, and vitamin B(12) levels in patients with systemic lupus erythematosus: A meta-analysis and meta-regression. J Am Coll Nutr (2021) 40(5):443–53. doi: 10.1080/07315724.2020.1788472 32702250

[10] Pan Y Liu Y Guo H Jabir MS Liu X Cui W. Associations between folate and vitamin B12 levels and inflammatory bowel disease: A meta-analysis. Nutrients (2017) 9(4):382. doi: 10.3390/nu9040382 PMC5409721 28406440

[11] Emdin CA Khera AV Kathiresan S. Mendelian randomization. Jama (2017) 318(19):1925–6. doi: 10.1001/jama.2017.17219 29164242

[12] Jin Y Andersen G Yorgov D Ferrara TM Ben S Brownson KM. Genome-wide association studies of autoimmune vitiligo identify 23 new risk loci and highlight key pathways and regulatory variants. Nat Genet (2016) 48(11):1418–24. doi: 10.1038/ng.3680 PMC5120758 27723757

[13] Liu JZ van Sommeren S Huang H Ng SC Alberts R Takahashi A. Association analyses identify 38 susceptibility loci for inflammatory bowel disease and highlight shared genetic risk across populations. Nat Genet (2015) 47(9):979–86. doi: 10.1038/ng.3359 PMC4881818 26192919

[14] Okada Y Wu D Trynka G Raj T Terao C Ikari K. Genetics of rheumatoid arthritis contributes to biology and drug discovery. Nature (2014) 506(7488):376–81. doi: 10.1038/nature12873 PMC3944098 24390342

[15] Bentham J Morris DL Graham DSC Pinder CL Tombleson P Behrens TW. Genetic association analyses implicate aberrant regulation of innate and adaptive immunity genes in the pathogenesis of systemic lupus erythematosus. Nat Genet (2015) 47(12):1457–64. doi: 10.1038/ng.3434 PMC4668589 26502338

[16] Grarup N Sulem P Sandholt CH Thorleifsson G Ahluwalia TS Steinthorsdottir V. Genetic architecture of vitamin B12 and folate levels uncovered applying deeply sequenced large datasets. PloS Genet (2013) 9(6):e1003530. doi: 10.1371/journal.pgen.1003530 23754956 PMC3674994

[17] Palmer TM Lawlor DA Harbord RM Sheehan NA Tobias JH Timpson NJ. Using multiple genetic variants as instrumental variables for modifiable risk factors. Stat Methods Med Res (2012) 21(3):223–42. doi: 10.1177/0962280210394459 PMC3917707 21216802

[18] Burgess S Butterworth A Thompson SG. Mendelian randomization analysis with multiple genetic variants using summarized data. Genet Epidemiol (2013) 37(7):658–65. doi: 10.1002/gepi.21758 PMC4377079 24114802

[19] Hemani G Bowden J Davey Smith G. Evaluating the potential role of pleiotropy in mendelian randomization studies. Hum Mol Genet (2018) 27(R2):R195–r208. doi: 10.1093/hmg/ddy163 29771313 PMC6061876

[20] Bowden J Davey Smith G Haycock PC Burgess S. Consistent estimation in mendelian randomization with some invalid instruments using a weighted median estimator. Genet Epidemiol (2016) 40(4):304–14. doi: 10.1002/gepi.21965 PMC4849733 27061298

[21] Hemani G Zheng J Elsworth B Wade KH Haberland V Baird D. The MR-base platform supports systematic causal inference across the human phenome. Elife (2018) 7:e34408. doi: 10.7554/eLife.34408 PMC5976434 29846171

[22] Zhu Z Zhang F Hu H Bakshi A Robinson MR Powell JE. Integration of summary data from GWAS and eQTL studies predicts complex trait gene targets. Nat Genet (2016) 48(5):481–7. doi: 10.1038/ng.3538 27019110

[23] Rodrigues M Ezzedine K Hamzavi I Pandya AG Harris JE. New discoveries in the pathogenesis and classification of vitiligo. J Am Acad Dermatol (2017) 77(1):1–13. doi: 10.1016/j.jaad.2016.10.048 28619550

[24] Chen J Zhuang T Chen J Tian Y Yi X Ni Q. Homocysteine induces melanocytes apoptosis *via* PERK-eIF2α-CHOP pathway in vitiligo. Clin Sci (Lond) (2020) 134(10):1127–41. doi: 10.1042/cs20200218 32400851

[25] Lerner AB Fitzpatrick TB. Biochemistry of melanin formation. Physiol Rev (1950) 30(1):91–126. doi: 10.1152/physrev.1950.30.1.91 15403662

[26] Shi W Meininger CJ Haynes TE Hatakeyama K Wu G. Regulation of tetrahydrobiopterin synthesis and bioavailability in endothelial cells. Cell Biochem Biophys (2004) 41(3):415–34. doi: 10.1385/CBB:41:3:415 15509890

[27] Schallreuter KU Kothari S Chavan B Spencer JD. Regulation of melanogenesis–controversies and new concepts. Exp Dermatol (2008) 17(5):395–404. doi: 10.1111/j.1600-0625.2007.00675.x 18177348

[28] Schallreuter KU. Advances in melanocyte basic science research. Dermatol Clin (2007) 25(3):283–91, vii. doi: 10.1016/j.det.2007.04.010 17662894

[29] Buglak AA Telegina TA Lyudnikova TA Vechtomova YL Kritsky MS. Photooxidation of tetrahydrobiopterin under UV irradiation: Possible pathways and mechanisms. Photochem Photobiol (2014) 90(5):1017–26. doi: 10.1111/php.12285 24773158

[30] Carreras-Torres R Ibáñez-Sanz G Obón-Santacana M Duell EJ Moreno V. Identifying environmental risk factors for inflammatory bowel diseases: A mendelian randomization study. Sci Rep (2020) 10(1):19273. doi: 10.1038/s41598-020-76361-2 33159156 PMC7648100

[31] Gibson RS. Principles of nutritional assessment. USA: Oxford university press (2005).

[32] Chanarin I. The megaloblastic anaemias. Oxford: Blackwell Scientific (1969).

[33] Adams JF Ross SK Mervyn L Boddy K King P. Absorption of cyanocobalamin, coenzyme b 12 , methylcobalamin, and hydroxocobalamin at different dose levels. Scand J Gastroenterol (1971) 6(3):249–52. doi: 10.3109/00365527109180702 5560708

[34] Scholten AM Vermeulen E Dhonukshe-Rutten RAM Verhagen T Visscher A Olivier A. Surplus vitamin B(12) use does not reduce fatigue in patients with irritable bowel syndrome or inflammatory bowel disease: A randomized double-blind placebo-controlled trial. Clin Nutr ESPEN (2018) 23:48–53. doi: 10.1016/j.clnesp.2017.10.004 29460813

[35] Benight NM Stoll B Chacko S da Silva VR Marini JC Gregory JF 3rd. B-vitamin deficiency is protective against DSS-induced colitis in mice. Am J Physiol Gastrointest Liver Physiol (2011) 301(2):G249–59. doi: 10.1152/ajpgi.00076.2011 PMC3154603 21596995

