# Supplementary material for: Genetically predicted levels of folate, vitamin B12, and risk of autoimmune diseases: A Mendelian randomization study
Source: Front Immunol. 2023 Mar 10;14:1139799. doi: 10.3389/fimmu.2023.1139799 (PMC10038229; doi:10.3389/fimmu.2023.1139799)
Supplement: Supplementary file 1 [file DataSheet_1.docx]

**Supplementary Material**

**Supplementary Table 1.** Detailed information of the genome-wide association studies in our analysis.

Abbreviations: IBD, Inflammatory bowel disease; RA, Rheumatoid arthritis; SLE, Systemic lupus erythematosus.

**Supplementary Table 2.** Detailed information for the SNPs in MR analysis.

Abbreviations: MR, Mendelian randomization; SNP, single nucleotide polymorphism; Chr, chromosome.

**Supplementary Table 3.** Details of variance explained by the selected instruments and F-statistics for the MR analysis based on the sample size of autoimmune diseases.

Abbreviations: OR, Odds Ratio.

**Supplementary Table 4.** Diseases and traits associated with genetic variants identified for folate and vitamin B_12_ at the genome-wide significance level.

**Supplementary Figure 1.** Plots of “leave-one-out” analysis for MR analysis of the causal effect of folate on vitiligo.

**Supplementary Table 1.** Detailed information of the genome-wide association studies in our analysis.

| Outcome | Year | PMID | Population | Sample size | |
| --- | --- | --- | --- | --- | --- |
|  |  |  |  | Cases | Controls |
| vitiligo | 2016 | 27723757 | European | 4680 | 39586 |
| IBD | 2015 | 26192919 | European | 38155 | 48485 |
| RA | 2014 | 24390342 | European | 14361 | 43923 |
| SLE | 2015 | 26502338 | European | 7219 | 15991 |
| Folate | 2013 | 23754956 | European | 37341 individuals | |
| Vitamin B_12_ |  |  |  | 45576 individuals | |

**Supplementary Table 2.** Detailed information for the SNPs in MR analysis

| Trait | SNP | Chr | Position | Nearest gene | Effect | exposure | | |  | Vitiligo | | |  | IBD | | |  | RA | | |  | SLE | | |
| --- | --- | --- | --- | --- | --- | --- | --- | --- | --- | --- | --- | --- | --- | --- | --- | --- | --- | --- | --- | --- | --- | --- | --- | --- |
|  |  |  |  |  | Allele | Beta | se | *P* |  | Beta | se | *P* |  | Beta | se | *P* |  | Beta | se | *P* |  | Beta | se | *P* |
| B_12_ | rs2270655 | 4 | 146576418 | TCN2 | C | 0.190 | 0.013 | 4.90×10^-49^ |  | 0.049 | 0.060 | 0.424 |  | -0.029 | 0.039 | 0.457 |  | -0.047 | 0.052 | 0.364 |  | 0.000 | 0.060 | 0.940 |
| B_12_ | rs1141321 | 6 | 49412433 | MUT | C | 0.061 | 0.006 | 3.60×10^-26^ |  | / | / | / |  | 0.016 | 0.017 | 0.358 |  | 0.032 | 0.020 | 0.109 |  | -0.030 | 0.030 | 0.259 |
| B_12_ | rs7788053 | 7 | 86773722 | TCN1 | G | 0.160 | 0.027 | 1.90×10^-9^ |  | / | / | / |  | / | / | / |  | / | / | / |  | 0.100 | 0.120 | 0.398 |
| B_12_ | rs1801222 | 10 | 17156151 | MMACHC | A | 0.510 | 0.086 | 3.00×10^-9^ |  | -0.010 | 0.030 | 0.854 |  | -0.004 | 0.018 | 0.815 |  | / | / | / |  | / | / | / |
| B_12_ | rs56077122 | 10 | 17207015 | CUBN | G | 0.110 | 0.006 | 3.30×10^-75^ |  | -0.010 | 0.030 | 0.627 |  | -0.016 | 0.018 | 0.356 |  | 0.018 | 0.021 | 0.398 |  | 0.000 | 0.030 | 0.965 |
| B_12_ | rs117456053 | 11 | 59616831 | MMAA | G | 0.066 | 0.009 | 2.20×10^-13^ |  | -0.010 | 0.130 | 0.911 |  | -0.063 | 0.079 | 0.423 |  | 0.059 | 0.046 | 0.198 |  | 0.020 | 0.060 | 0.684 |
| B_12_ | rs12272669 | 11 | 71392610 | CD320 | T | 0.320 | 0.020 | 8.40×10^-59^ |  | / | / | / |  | 0.043 | 0.057 | 0.453 |  | -0.025 | 0.055 | 0.648 |  | 0.050 | 0.070 | 0.482 |
| B_12_ | rs34324219 | 11 | 59623378 | TCN1 | C | 0.210 | 0.009 | 1.10×10^-111^ |  | -0.010 | 0.050 | 0.801 |  | -0.048 | 0.030 | 0.112 |  | -0.069 | 0.056 | 0.215 |  | -0.060 | 0.040 | 0.169 |
| B_12_ | rs34528912 | 11 | 59631535 | TCN1 | T | 0.170 | 0.021 | 2.10×10^-15^ |  | 0.095 | 0.070 | 0.190 |  | 0.011 | 0.054 | 0.834 |  | / | / | / |  | 0.070 | 0.070 | 0.282 |
| B_12_ | rs41281112 | 13 | 100518634 | ABCD4 | T | 0.045 | 0.006 | 1.70×10^-13^ |  | 0.086 | 0.120 | 0.475 |  | 0.166 | 0.081 | 0.041 |  | -0.017 | 0.020 | 0.410 |  | -0.040 | 0.030 | 0.163 |
| B_12_ | rs3742801 | 14 | 74759006 | CLYBL | C | 0.170 | 0.014 | 8.90×10^-35^ |  | 0.000 | 0.030 | 0.917 |  | -0.020 | 0.017 | 0.254 |  | 0.106 | 0.074 | 0.152 |  | / | / | / |
| B_12_ | rs2336573 | 19 | 8367709 | CUBN/TRDMT1 | A | 0.087 | 0.009 | 4.80×10^-21^ |  | 0.068 | 0.080 | 0.406 |  | 0.048 | 0.045 | 0.290 |  | 0.020 | 0.016 | 0.235 |  | 0.020 | 0.030 | 0.413 |
| B_12_ | rs602662 | 19 | 49206985 | TCN2 | C | 0.064 | 0.010 | 7.50×10^-10^ |  | 0.020 | 0.030 | 0.588 |  | 0.062 | 0.017 | 0.000 |  | -0.006 | 0.019 | 0.751 |  | -0.050 | 0.030 | 0.120 |
| B_12_ | rs1131603 | 22 | 31018975 | FUT2 | A | 0.160 | 0.006 | 2.40×10^-139^ |  | -0.073 | 0.070 | 0.289 |  | 0.003 | 0.040 | 0.949 |  | 0.005 | 0.017 | 0.780 |  | 0.110 | 0.030 | 0.000 |
| B_12_ | rs5753231 | 22 | 31003069 | FUT6 | A | 0.046 | 0.007 | 1.70×10^-10^ |  | -0.030 | 0.040 | 0.407 |  | -0.015 | 0.021 | 0.457 |  | / | / | / |  | / | / | / |
| folate | rs5753231 | 1 | 11856378 | MTHFR | G | 0.096 | 0.006 | 9.50×10^-53^ |  | 0.104 | 0.030 | 0.001 |  | 0.005 | 0.022 | 0.812 |  | 0.003 | 0.020 | 0.894 |  | -0.070 | 0.040 | 0.074 |
| folate | rs17421511 | 1 | 11857788 | MTHFR | G | 0.098 | 0.012 | 1.80×10^-15^ |  | 0.020 | 0.040 | 0.630 |  | 0.021 | 0.018 | 0.236 |  | -0.014 | 0.017 | 0.419 |  | 0.040 | 0.030 | 0.163 |
| folate | rs1999594 | 1 | 11959216 | MTHFR | A | 0.076 | 0.008 | 7.50×10^-20^ |  | -0.051 | 0.030 | 0.064 |  | -0.030 | 0.017 | 0.074 |  | 0.028 | 0.015 | 0.069 |  | 0.040 | 0.030 | 0.126 |
| folate | rs652197 | 11 | 71849741 | FOLR3 | C | 0.069 | 0.010 | 1.40×10^-12^ |  | -0.062 | 0.040 | 0.184 |  | -0.054 | 0.026 | 0.040 |  | 0.016 | 0.034 | 0.644 |  | -0.020 | 0.040 | 0.580 |

**Supplementary Table 3.** Details of variance explained by the selected instruments and F-statistics for the MR analysis based on the sample size of autoimmune diseases.

| **exposure** | **Outcome** | **case** | **control** | **R2^*^of instrument** | **F-statistic median**  **(range)** | **OR**^†^ **estimated for α<0.05 and power≥80%** | **OR**^†^ **estimated for α<6.25×10^-3^ (Bonferroni adjustment) and power ≥ 80% with Bonferroni adjustment** |
| --- | --- | --- | --- | --- | --- | --- | --- |
| B_12_ | vitiligo | 4680 | 39586 | 0.049 | 142.22 (22.05-568.86) | 1.199 | 1.254 |
| Folate | vitiligo | 4680 | 39586 | 0.011 | 104.86 (52.32-153.26) | 1.424 | 1.550 |
| B_12_ | IBD | 38155 | 48485 | 0.052 | 111.04 (22.05-568.86) | 1.087 | 1.185 |
| Folate | IBD | 38155 | 48485 | 0.011 | 104.86 (52.32-153.26) | 1.196 | 1.429 |
| B_12_ | RA | 14361 | 43923 | 0.048 | 154.21 (22.05-568.86) | 1.126 | 1.163 |
| Folate | RA | 14361 | 43923 | 0.011 | 104.86 (52.32-153.26) | 1.270 | 1.350 |
| B_12_ | SLE | 7219 | 15991 | 0.048 | 123.03 (22.05-568.86) | 1.190 | 1.246 |
| Folate | SLE | 7219 | 15991 | 0.011 | 104.86 (52.32-153.26) | 1.420 | 1.548 |

*$\text{R}^{\text{2}}\text{=}{\text{(}\text{β×}\sqrt{\text{2×MAF}\left( \text{1-MAF} \right)}\text{)}}^{\text{2}}$.β: effect size estimates of the SNPs of vitamin B_12_ and folate levels; MAF: minimum allele frequency.

† The odds ratio per 1SD elevate in vitamin B_12_ or folate levels, for which there is power (1-β) ≥80% to detect an existed association at α<0.05.

**Supplemental Table 4.** Diseases and traits associated with genetic variants identified for folate and vitamin B_12_ at the genome-wide significance level

| SNP | *P*-value | Trait(s) | PubMed ID | Location |
| --- | --- | --- | --- | --- |
| **vitamin B_12_** |  |  |  |  |
| rs1801222 | 2×10^-13^ | stroke | 25147783 | 10:17114152 |
|  | 2 ×10^-13^ | body height | 30595370 | 10:17114152 |
| rs34324219 | 5 ×10^-11^ | stroke | 25147783 | 11:59855905 |
|  | 3×10^-25^ | blood protein measurement | 29875488 | 11:59855905 |
| rs34528912 | 5×10^-30^ | Blood protein levels | 29875488 | 11:59864062 |
| rs5753231 | 8×10^-13^ | blood protein measurement | 29875488 | 22:30607082 |
| rs602662 | 5×10^-8^ | autoimmune thyroid disease, type I diabetes mellitus, common variable immunodeficiency, ankylosing spondylitis, psoriasis, celiac disease, ulcerative colitis, Crohn's disease, autoimmune disease, juvenile idiopathic arthritis, systemic lupus erythematosus | 26301688 | 19:48703728 |
| **folate** |  |  |  |  |
| rs1801133 | 6×10^-9^ | high altitude adaptation | 28373541 | 1:11796321 |
|  | 1×10^-12^ | mean reticulocyte volume | 32888494 | 1:11796321 |
|  | 2×10^-15^ | multiple sclerosis | 31604244 | 1:11796321 |
|  | 1×10^-23^ | red blood cell distribution width | 30595370 | 1:11796321 |

**
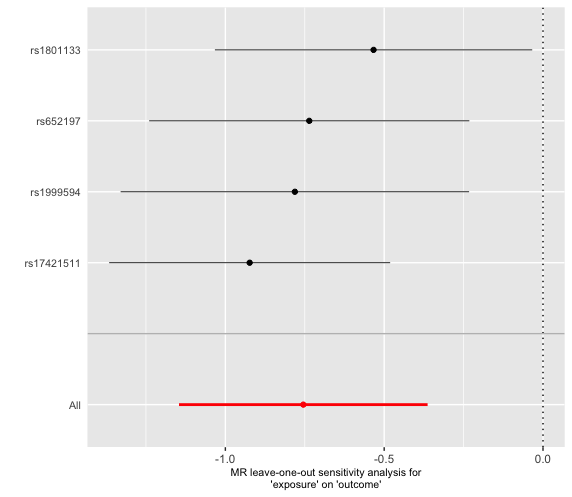
**

**Supplementary Figure 1.** Plots of “leave-one-out” analysis for MR analysis of the causal effect of folate on vitiligo.
